# A Logic Model for Evaluation and Planning in an Adult Day Care for Disabled Japanese Old People

**DOI:** 10.3390/ijerph17062061

**Published:** 2020-03-20

**Authors:** Takashi Naruse, Ayaka Kitano, Hiroshige Matsumoto, Satoko Nagata

**Affiliations:** 1 Department of Community Health Nursing, Graduate School of Medicine, The University of Tokyo, 7-3-1 Hongo, Bunkyo-ku, Tokyo 113-0033, Japan; pb.hiroshige@gmail.com; 2 School of Nursing, Midwifery & Social Work, The University of Queensland, At Lucia, QLD 4072, Australia; 3 Analytics & Innovation Department, Business Development Division, SMS Co., Ltd., 2-11-1 Shibakouen, Minato-ku, Tokyo 105-0011, Japan; ayaka0408cooky@gmail.com; 4 Faculty of Nursing and Medical Care, Keio University, 4411 Endo, Fujisawa, Kanagawa 252-0883, Japan; satokon@sfc.keio.ac.jp

**Keywords:** adult day care, group interview, logic model development, quality management

## Abstract

Adult day care (ADC) provides various services for meeting clients’ needs. Based on the mini-Delphi method with 46 ADC staff and the discussions with eight ADC administrators, this study developed and finalized a comprehensive logic model to represent the elements of ADC. For the three basic structures of a logic model—inputs/activities, outputs, and outcomes—the model contained seven core categories and 23 sub-categories. The ADC inputs/activities consisted of two core categories: “Place to stay” and “Intervention from staff”. These inputs/activities caused two kinds of outputs: “Clients’ experiences” and “Families’ experiences”. “Accumulating experiences” with repeated ADC visits was established as the link between the ADC outputs and outcomes, which were “Clients’ change” and “Families’ change”. ADC centers provide various experiences for their clients and their caregivers, ranging from the fulfillment of needs for fundamental care to psychiatric care and self-actualization. Improving various model-related inputs/interventions can produce better experiences and outcomes. The model can guide ADC administrators, policymakers, and researchers in the evaluation of a heterogeneous ADC service program that is community-based, thereby ensuring optimal care for clients with an efficient use of resources.

## 1. Introduction

With rapidly aging global populations, the disabled aged population has increased. Long-term care (LTC) is one of the approaches which provides health promotion for disabled persons, and expenditures on long-term care (LTC) have increased. As a share of the gross domestic product (GDP), LTC increased more rapidly than any other health care expenditure during the 2005–2015 period [1]. An additional consequence of aging populations is the increased proportion of older people living at home [1]. In Japan, younger generations have responded to the need to spend a greater proportion of Japan’s GDP on LTC by becoming hyper-vigilant regarding the “whats” and “hows” of the care being offered to older people. Increased spending on LTC, coupled with the vigilance of the young, has contributed to a growing interest in adult day care (ADC) services/centers for the disabled elderly population. With such economic and societal “pressures”, ADC staff must provide optimal care while maintaining an efficient use of resources.

In Japan, ADC is one of the LTC services. About 12% of the aged population in Japan used one or more LTC services in April 2018. National LTC insurance covered LTC services, and disabled persons (over 97% of them were 65 years or over) can use the LTC services under insurance fee coverage [2]. The number of ADC clients was 1.13 million (in April 2018, about 26.8% of the disabled population), and ADC was the most popular service among the disabled population [2]. One international review demonstrated that ADC providers had four general aims: (i) providing social and preventive services, (ii) supporting clients’ continued independence, (iii) supporting attendees’ health and daily living needs, and (iv) enabling family members to take a break from caregiving and/or continue with employment [3]. For ADC clients, the intervention-related benefits included improved physical, mental, and social function, exposure to comprehensive care, and the alleviation of caregivers’ burdens [3,4,5].

Due to the client and staff heterogeneity in ADCs, it is difficult to objectively demonstrate the program and intervention benefits [6]. In this study, we propose a logic model as an effective way to evaluate a heterogeneous program. The definition of the logic model was explained as “a picture of how you believe your program will work. It uses words and/or pictures to describe the sequence of activities thought to bring about change and how these activities are linked to the results the program is expected to achieve” [7]. A logic model diagram is a systematic chart showing the following program elements: resource inputs, activities, outputs, resulting outcomes, and the impacts to be achieved [7]. Scrutinizing the model’s elements helps to better understand and evaluate the targeted inputs under complex intervention conditions. Subirana et al. used a logic model to elucidate the possible ways in which nurse staffing can affect patient and nurse outcomes [8]. In school nursing, a logic model was reported to be useful in building a stakeholder consensus for interventions targeting teen pregnancy prevention [9]. In long-term care settings, logic models were developed before the development of the interventions/guidelines for pressure ulcer prevention within nursing homes [10,11]. Based on the usefulness of the logic models in previous research, this study aimed to develop a logic model for ADC in Japan.

Previous research has explored the relationships between the component elements of ADC. Some studies indicated that clients’ experiences mediated between the ADC interventions and the outcomes for clients and family caregivers. A literature review of ADC effectiveness (2000–2011) [5] integrated pieces of evidence into a basic logic model and revealed that ADC use was related to clients’ service-related experiences and caregivers’ use of respite care. However, it remained unclear what the ADC program’s elements of inputs/activities were, as well as the relationships among the elements.

Elsewhere, Gaugler described ADC usage and developed a measurement scale for evaluating the service process underlying the outcome performance [12,13]. Considering the increase in the aging population and the vigilance of the younger generation in Japan, it is important to provide optimal experiences for clients despite the fewer service inputs. Before explaining the cause and effect in relation to the inputs and outcomes in the current study, we first investigated the nature of each of ADC’s component elements.

## 2. Materials and Methods

Our methodological approach was based on two previous studies in which the researchers developed a logic model for their interventions [14,15]. In the first step, the model elements were extracted based on the inputs of ADC staff, using the mini-Delphi method. In the second step, which occurred only after the element items were determined, we consulted with ADC administrators to (re)construct the items and refine the emerging logic model. In the third step, ADC staff reviewed the emerging model for final refinement purposes. The relevant institutional board approved this study.

### 2.1. Mini-Delphi Method

Five Tokyo ADC facilities were selected through snowball sampling [16]. All facility administrators agreed to participate in a group interview using the mini-Delphi method. We provided the materials explaining the research to each facility, and 46 staff (5–15 staff in each facility: 27 certified care workers, five nurses, five social workers, five occupational therapists, three administrators, and one dental hygienist) agreed to participate and provided written consent.

Group interviews were conducted at each facility during the months June–September in 2016. The researchers visited each facility and asked the staff to participate in the group interviews. The participants sat around a table, and one facilitator–researcher stood in front of them. At the beginning of the interview, we asked the participants to list up to eight items regarding “interventions and experiences provided at your ADC facility.” Furthermore, the participants were asked to list items in response to “What do your clients achieve?” and “Why do your clients come to ADC, and what do they do?”. To mitigate the “group-think” [17], Post-It NotesTM (3M Japan, Tokyo, Japan) were used to gather the individuals’ opinions.

The researchers collected the listed items on a large whiteboard, and, together with the participants, they classified them by similar meanings. First, the researchers asked each participant to post their notes on the whiteboard. Items with the same meaning as those already attached were placed closer together. One researcher facilitated the process and confirmed the meaning of the items which were already attached to the participant posting them. Each participant also voiced their observations as they were posting their notes and corrected the notes already posted on the whiteboard. If a participant thought of a new list item while organizing, they recorded it on an additional note. Two facilitator–researchers spoke in lieu of the participants as they were posting their notes, allowing the participants to focus on the classification process. At the end of the interview, the researchers confirmed the names and meanings of all the listed items. Each group interview lasted between 60 to 90 min. A total of five group interviews were implemented across the five facilities. The researchers then obtained the preliminary categorizations of each of the five interviews’ results. A researcher transcribed the spoken contents of the interviews.

### 2.2. Administrator Discussions

In December 2016, three meetings were conducted with the administrators from three ADC facilities to integrate the preliminary categorized results of each group interview. The researchers ensured that the meaning of the original data was not changed during the integration by the administrators. The first meeting focused on discussing the thoughts and ideas from the group interviews. Before the meeting, the preliminary categorized results of each group interview were developed by the researchers. The researchers entered all the names and meanings of the listed items from the five interviews into one table. A set of names and meanings was put in the same row. All rows were sorted by “name”, and duplications of the same name were colored with a red marker. We showed the table to the administrators. Together, the researchers and administrators categorized the items according to their similarities and differences. Then, the five results were integrated into one table which included the names and meanings of the listed items. The same three administrators participated in the second meeting, and the discussion focused on classifying the items into sub-categories to show the cause and effect regarding ADC attendance. The names of the sub-categories were extracted from the names of the listed items. The relationships between the sub-categories were discussed, and the sub-categories were sorted according to the core categories as key components of a comprehensive logic model. Eight administrators from seven ADCs (including the three from the first and second discussions) participated in the third discussion, in which the logic model from the second discussion was checked and modified. Each discussion lasted between 60 and 120 min. The administrators provided written consent for their participation in all three discussions.

### 2.3. Model Checking and Feedback from ADC Staff and Clients

To assess the model’s validity, we solicited the feedback of the 46 ADC staff who had attended an ADC skill-up seminar organized by a Tokyo social welfare organization in 2017. The first researcher explained the developed model to the participants and asked for their feedback to be given directly to the researcher during the seminar.

## 3. Results

### Summary of Finalized Logic Model

**Figure 1:**
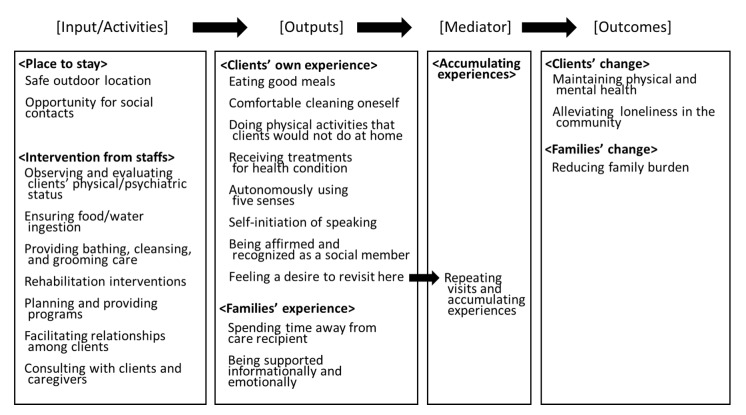
Developed Logic Model of Adult Day Care Service. Shown are the categories and sub-categories for the logic model of adult day care services. Categories (shown by <…>) and sub-categories are shown. Arrows indicate “cause and effect”.

The finalized model contained seven core categories and 23 sub-categories (Figure 1). Table 1 shows the sub-categories and item examples for each sub-category. A total of 101 items were extracted from the group interviews and discussions. The items were divided into three categories: inputs/activities, outputs, and outcomes. The inputs/activities referred to the equipment and interventions within ADCs. Within inputs/activities, the “Place to stay” category concerned the geographic aspect of ADCs, where clients can be safe and surrounded by people, whereas the “Intervention from staff” category concerned the interventions and behaviors by ADC staff. The outputs concerned the results of the inputs and activities. The “Clients’ own experience” and “Families’ experience” categories concerned the events experienced by the clients/families when they stayed at ADCs. It included all the clients’ experiences in reaction to the ADC inputs/activities. The “Outcomes” category concerned the changes or benefits that resulted from ADC.

During the third discussion with the administrators, the “Accumulating experiences” category was added between the outputs and outcomes to explain how the outputs relate to the outcomes. It specified one way in which the use of ADC related to the three outcome types; that is, through the accumulation of repeated visits. Furthermore, through the “Feeling a desire to revisiting here” output sub-category, clients’ experiences at the ADC were expected to cause an accumulation of experiences. The model suggested that ADCs produced positive outcomes when clients continuously received professional intervention in a safe environment that provided them with opportunities for social interaction.

Despite several discussions with the administrators, they could not connect sub-categories with arrows for explaining cause and effect. The administrators explained that each sub-category included multiple meanings, and the direction of cause and effect was complex. Because the objective of this model was to explain the nature of ADC’s component elements, precise meanings were not developed for the sub-categories, and arrows for explaining cause and effect were connected mainly between the categories. When the model was checked with the 46 ADC staff, they recommended no modifications to the model. One participant administrator (id1) said:

*When we provide a meal, the first meaning that one thinks of is “sufficient ingestion for nutrition.” But I believe that the opportunity to eat a meal with other companions provides a sense of relationship with others. It might require one old woman to use all five senses for good conversation, including speaking on her own initiative. To ensure she has the opportunity for these experiences, there is a need to ensure she ingests food with staff’s careful observation and the facilitation of clients. Combinations [of the items] within an intervention happen all the time and everywhere within a center. Of course, we are trying to provide every multi-layered experience to meet clients’ needs because it will relate to better change (for clients and family caregivers).*

## 4. Discussion

We described a logic model for ADC that has two service aspects, namely “a place to stay” and “ntervention from staffs”, and which provides various experiences for clients and their caregivers, ranging from the fulfillment of needs for fundamental care to psychiatric/self-actualization. The described model makes two contributions to the understanding of the cause and effect within ADC services. First, the model clarifies the elements of the model components. Second, the accumulation of experiences was determined to be an antecedent of the outcome improvement.

The logic model for the ADC elements was developed with the direct input of ADC staff. Improving various model-related inputs/interventions is expected to produce better experiences and outcomes for both clients and their caregivers. Previous researchers have examined the effects of the numerous interventions specified in the model on the clients’/family caregivers’ outcomes. These interventions include rehabilitation [18], physical activities [19], art programs [20], education or skills training [21], socialization [22], and family support and counseling [23]. Our model indicates that there are certain gaps in the research on inputs and interventions, which include “safe outdoor location”, “opportunity for social contacts”, “observing and evaluating clients’ physical/psychiatric status”, “ensuring food/water ingestion”, and “providing bathing, cleansing, and grooming care”.

The model’s indicated inputs/activities include having a place to stay and staff intervention. Staff intervention can take place in the home through home-visiting services and other home-based care services, but having a place to stay is a distinctive ADC component. Spatial separation from the home allows family caregivers to “spend time away from the care recipient” and provides a “safe outdoor location” for clients. The social environment may be one reason why the staff interventions improve clients’ experiences. Positive interventions and/or program outcomes could be attributable to the opportunities provided by ADCs to interact socially in a group setting [24]. ADC was reported to buffer isolation through the maintenance and development of social relationships and access to the world through activities [25]. Because all interventions must be conducted in a safe environment, ADC staff may be required to provide closer attention and more intensive support to clients with more severe difficulties. However, the clients may consider a too-supportive environment infantilizing. Thist can prevent or interfere with their autonomy and social interactions [26], which are the expected outputs in the logic model for ADC. To maximize the ADC benefits, the design of the inputs/activities should consider the relationships between the clients’ abilities and needs on the one hand, and the interventions and their environments on the other hand. For example, among elderly female patients with dementia who have good knitting skills, knitting activities facilitated better conversations and experiences [27]. Future research is necessary to explain how a person–environment fit leads to positive ADC outcomes.

To ensure the achievement of the goals and outcomes for each client and their family caregiver, the client’s accumulated experience, that is, their access to repeated and longitudinal interventions, is essential. The model found that “Feeling a desire to revisit here” is a fundamental element for ensuring the accumulated experience in ADC. The interventions aimed at maintaining clients’ motivations with regard to ADC could contribute to ensuring the ADC benefits.

The three extracted outcomes in the logic model are considered to be an achievement of the four general ADC aims identified by Orellana et al. [3]: “Maintaining physical/mental health” reflects the goal of supporting clients’ continued independence, “supporting attendees’ health and daily living needs” and “Alleviating loneliness” reflects the goal of providing social and preventive services, and “Reducing family burden” reflects enabling family caregivers to take a break and/or continue with employment. The logic model suggests that improvements in these outcomes are the expected result of the accumulated diverse need fulfillments with continuous ADC use. Our model also describes how ADC could produce positive changes among clients and their family caregivers, which is a result that has been demonstrated by Zarit [28] and other researchers.

One unexpected yet interesting result arose from the discussion with administrators on the difficulty of connecting sub-categories with arrows for explaining cause and effect. It was explained that multiple interventions were provided simultaneously to one client. For example, sitting together with other clients during mealtimes can be considered an input/activity corresponding to “Opportunity for social contacts” because of the interaction with others at the table, “Ensuring food/water ingestion” because of the provided food, and “Rehabilitation intervention” because of the use of chopsticks. The clients could also experience “Eating good meals” because of the food’s nutritional value, “Autonomously using five senses” because of the tasting of the food and the listening to others, and “Self-initiation of speaking” because of the conversation that would occur. Since eating with others can increase energy intake relative to eating alone, social isolation and malnutrition are closely linked [29]. An ADC program like social mealtimes might have a greater impact by combining multiple inputs/activities compared with one that does not. This could be an important benefit of ADC with diverse inputs/activities, and future research should focus not only on the effect of each independent input/activity but also on the combinations of the ADC inputs/activities.

ADC was explained to contribute to clients’ function and health. The use of ADC might promote health among the disabled aged population. If an old person is physically or psychiatrically disabled in daily life, it would be difficult for them to participate in general health promotion activities/interventions for independent persons. The ADC inputs/activities might meet the diverse needs of disabled persons, and help him/her to receive the benefits of ADC. Once he/she visits an ADC center, service professionals should ensure he/she feels a desire to revisit. This could ensure that health promotion for clients is achieved.

This research has some limitations. To maintain the feasibility of the interviews and the transformability of the results for the ADC clients with a diverse range of disabilities, the current model was developed based only on interviews with ADC staff. In Japan, the percentage of ADC clients with dementia was found to be 52% [30], and about 30%–40% of ADC clients were estimated to live by themselves by the ADC administrators who participated in this study. This condition limited the possibility of interviewing both clients and family caregivers. Because the model also suggests that clients and family caregivers are the significant ADC beneficiaries, the perspective of caregivers must be reflected in the evaluation of the service experience. In addition, the quality of ADC offered to disabled older adults may have consequences for their healthcare and LTC services. Future research should include the perspectives of the clients, family caregivers, and other service providers in finalizing the model for evaluating the service process, which could help to improve the quality of the future of ADC services.

## 5. Conclusions

In this study, the logic model for ADC was developed based on the perspectives of ADC staff. The model contained four elements regarding the ADC service process, seven core categories, and 23 sub-categories. The four elements were inputs/activities, outputs, outcomes, and a mediator between outputs and outcomes. The inputs/activities consisted of two core categories: “Place to stay” and “intervention from staff”. The inputs/activities led to two core categories of outputs: “Clients’ experiences” and “Families’ experiences”. The core category of “Accumulating experiences” with repeated ADC visits mediated the relationship between the outputs and the two core categories of the outcomes of ADC services, namely “Clients’ change” and “Families’ change”.

ADC provides various experiences for clients and their caregivers, ranging from the fulfillment of needs for fundamental care to psychiatric/self-actualization. Improving various model-related inputs/interventions is expected to produce better experiences and outcomes. The logic model developed in this study can guide ADC administrators, policymakers, and researchers in the evaluation of a heterogeneous ADC program which serves the community, thereby ensuring optimal care with an efficient use of resources.

## Figures and Tables

**Table 1:** Sub-Categories and Item Examples.

Sub-Categories	Item Examples
Safe outdoor location	Bus transportation for safety Regular schedule for going outdoors and on trips Adequate respite care
Opportunity for social contacts	Being in the company of others Contact with other people in public spaces
Observing and evaluating clients’ physical/psychiatric status	Assessment of physical and psychiatric status
Ensuring food/water ingestion	Ensuring food and water ingestion
Providing bathing, cleansing, and grooming care	Bathing care Cleansing and grooming Toothbrushing
Rehabilitation intervention	Rehabilitation training and exercise
Planning and providing programs	Exercise program Arts and crafts program Seasonal events
Facilitating relationships among clients	Seating arrangement Grouping clients who have good relationships with each other Facilitating clients’ conversations
Consulting with clients and caregivers	Consulting with clients Consulting with family caregivers
Eating good meals	Meals with good nutrition Comfortable meal time Self-assessing nutrition status
Comfortable cleaning oneself	Cleaning oneself and feeling comfortable Cleaning oneself in a safe environment and an appropriate manner
Doing physical activities that clients would not do at home	Exercising appropriately under supervision and in a safe environment Physically active in the daytime Exercising unconsciously in group activities
Receiving treatments for health condition(s)	Receiving assessments of physical and psychiatric status Knowing one’s own health condition(s) Receiving medical and nursing treatment Being referred to appropriate medical facilities
Autonomously using five senses	Autonomously using five senses
Self-initiation of speaking	Actively communicating with others without prompting Selecting ideas or thoughts to express to others
Being affirmed and recognized as a social member	Recognizing one’s own value in helping others Being recognized in a public setting Recognizing one’s own place in the world
Feeling a desire to revisit here	Feeling a desire to revisit
Spending time away from care recipient	Taking rest while attendee is at ADC Completing daily work while the older adult is at ADC
Being supported informationally and emotionally	Obtaining information about the physical/psychiatric condition of clients Having a consultation with ADC staff
Repeating visits and accumulating experiences	Repeating visits and accumulating experiences
Maintaining physical and mental health	Maintaining cognitive function Maintaining wellness in physical functioning Improving and maintaining ADL
Alleviating loneliness in the community	Preventing social withdrawal Developing human relationships/friendships with others Developing and maintaining social skills
Reducing family burden	Reducing the caregiving burden on the family Alleviating family anxiety about care recipient’s disability

Note. ADC is adult day care; ADL is activities of daily living.
